# Video-assisted thoracic surgery and thoracic empyema

**DOI:** 10.4103/0970-2113.76315

**Published:** 2011

**Authors:** Nikolaos Barbetakis, Christos Asteriou, Christodoulos Tsilikas

**Affiliations:** *Department of Thoracic Surgery, Theagenio Hospital, Al. Simeonidi, Thessaloniki, Greece E-mail: nibarbet@yahoo.gr*

Sir,

We read with great interest Kundu and colleagues remarkable experience with the surgical management of tuberculous and nontuberculous thoracic empyema and we would like to congratulate them.[1] The role of surgical intervention especially in tuberculosis has decreased dramatically with the advent of powerful antituberculous drugs. However, it would be very useful to know the exact stage (I, II or III) of the disease in order to assess the efficacy of each method used (fibrinolysis, VATS, and open thoracotomy). Another point that has to be highlighted is the absence of video-assisted thoracic surgery in the treatment of thoracic empyema.

A propos of this comment, we would like to share our institute’s surgical experience with the disease, which could be summarized in the following points: The use of fibrinolysis is absolutely indicated for stages I and II (exudative and fibrinopurulent stages, respectively). The use of VATS seems to be effective for stage II empyemas. Open thoracotomy and decortication are the procedure of choice for stage III empyemas (organised fibrothorax).

Minimally invasive techniques such as VATS are increasingly used and provide a new approach to the management of thoracic empyema.[2] Concerning tuberculous empyemas extreme narrowing of the intercostal spaces and thickened parietal peel make introduction of the thoracic ports and manipulation of the endoscopic instruments very difficult. Thus assessment of and judgment as to whether an optimal peeling has been gained without causing excessive air leak are difficult via thoracoscope and consequently require extreme experience.[3] It is certain that the increased and ongoing experience with VATS will allow us to perform more sophisticated procedures in the near future.
